# Beyond Technological Advances: Additional Considerations Regarding Temporal Trends in Atrial Fibrillation Ablation

**DOI:** 10.1002/clc.70357

**Published:** 2026-05-25

**Authors:** Naoya Kataoka, Teruhiko Imamura

**Affiliations:** ^1^ Second Department of Internal Medicine University of Toyama Toyama Japan

To editor,

We read with great interest the recent article, investigating temporal trends in atrial fibrillation (AF) catheter ablation (CA) procedures in Germany between 2016 and 2022 using a large multicenter administrative database [1]. The authors demonstrated a steady increase in AF ablation procedures despite the temporary disruption caused by the COVID‐19 pandemic, together with reductions in transesophageal echocardiography (TEE) utilization, intensive care treatment, and length of hospital stay. Several issues warrant further discussion.

The mechanisms underlying the increasing number of AF ablation procedures may be more complex than technological progress alone. The authors appropriately discussed improvements in ablation technologies and expansion of indications for CA, particularly following evidence supporting CA in heart failure populations [1]. However, healthcare policy and reimbursement systems may also substantially influence procedural trends [2]. For example, diagnosis‐related group–based reimbursement structures may incentivize high procedural volumes and shorter hospitalizations, particularly for procedures such as AF ablation that are generally completed during short inpatient stays [3]. In addition, expansion in the number of electrophysiology centers and aging of the general population with increasing AF prevalence may also contribute to the observed increase in procedure numbers. Accordingly, the increase in AF ablation utilization should perhaps be interpreted not only from a clinical perspective, but also from the standpoint of healthcare economics and administrative policy.

The observed reduction in hospital length of stay may not be explained solely by procedural streamlining and technological advancement. Economic pressures to shorten hospitalization may also have contributed to this trend. Under fixed reimbursement systems, shorter hospitalization durations are often associated with improved hospital efficiency and profitability [4]. Thus, discharge timing may partly reflect institutional operational strategies in addition to purely medical considerations.

The substantial decline in TEE utilization is intriguing and deserves additional clarification. Although contemporary anticoagulation strategies and risk stratification may allow omission of routine preprocedural TEE in selected low‐risk patients, low CHA_2_DS_2_‐VASc scores do not necessarily exclude the presence of left atrial appendage thrombus [5]. In real‐world practice, another potential explanation may be limited institutional TEE resources, particularly because structural heart interventions increasingly compete for echocardiographic laboratory capacity. Additional discussion regarding procedural selection criteria for TEE omission would therefore enhance interpretation of these findings.

## Funding

The authors have nothing to report.

## Ethics Statement

The authors have nothing to report.

## Consent

The authors have nothing to report.

## Conflicts of Interest

The authors declare no conflicts of interest.

## Data Availability

Data sharing not applicable to this article as no datasets were generated or analyzed during the current study. The manuscript does not include any original data.
